# Prevalence and risk factors for patent *Toxocara* infections in cats and cat owners’ attitude towards deworming

**DOI:** 10.1007/s00436-016-5242-8

**Published:** 2016-09-17

**Authors:** R. Nijsse, H. W. Ploeger, J. A. Wagenaar, L. Mughini-Gras

**Affiliations:** 1 Department of Infectious Diseases and Immunology, Faculty of Veterinary Medicine, Utrecht University, P.O.Box 80.165, 3508 TD Utrecht, The Netherlands; 2 National Institute for Public Health and the Environment, Centre for Infectious Disease Control, P.O. Box 1, 3720 BA Bilthoven, The Netherlands; 3 Central Veterinary Institute of Wageningen UR, Houtribweg 39, 8221 RA Lelystad, The Netherlands

**Keywords:** *Toxocara cati*, Household cats, Risk factors, Deworming, Cat owners, Public health

## Abstract

The prevalence of and risk factors for shedding *Toxocara* eggs in cats older than 6 months were determined by examining 670 faecal samples collected in 4 cross-sectional studies in the Netherlands. Additionally, cat owners provided information on their attitude towards routine deworming. Samples were examined using the centrifugal sedimentation flotation method. Overall *Toxocara* prevalence was 7.2 %. Multivariable logistic regression analysis revealed that young age and living in rural areas were significant risk factors for shedding *Toxocara* eggs. Moreover, the more time a cat was allowed to roam outdoors, the higher was its risk to shed *Toxocara* as compared to cats with no outdoor access at all. For 199 cats (81.6 % of cats subjected to a deworming regimen) owners provided the reason for treatment. The main reason for routine deworming (80.4 %) concerned the cat’s health and only 10.6 % of the cats were treated for public health reasons. Moreover, the generally advocated four-times-a-year deworming advice was applied on only 24.5 % of cats. We concluded that free roaming is a key factor in the acquisition of patent *Toxocara* infections leading to the environmental contamination with *Toxocara* eggs. Additionally, the knowledge of cat owners is still insufficient to expect them to make sound decisions on routine deworming.

## Introduction

Cats are among the most common pets worldwide, and in a country like the Netherlands, their estimated number is almost twice as large as that of dogs (HAS den Bosch and Utrecht University 2015). Additionally, while the Netherlands is a country free of stray dogs, stray, and free-ranging cats are widespread (Neijenhuis and van Niekerk 2015). These unowned cats are more likely to receive sub-optimal care and potentially harbour more parasites.

*Toxocara cati* is a zoonotic roundworm of cats that is known to commonly affect both well-cared and stray cats. Compared to its congeneric species *Toxocara canis* in dogs, the epidemiology of *T. cati* is more unclear (Fisher 2003). However, among adult hosts of *Toxocara* spp. in the Netherlands, i.e. cats, dogs, and foxes, cats have been estimated to be responsible for a considerable, if not the largest, portion of *Toxocara* spp. eggs contaminating the environment (Morgan et al. 2013; Nijsse et al. 2015). With the aim of reducing environmental contamination with *Toxocara* eggs, the guidelines of the European Scientific Counsel Companion Animal Parasites (ESCCAP) state that all adult cats should be dewormed at least four times a year to prevent patent *T. cati* infections (ESCCAP 2010). However, the compliance of cat owners to this advice is unlikely to be high enough to have a significant impact on the environmental contamination with *Toxocara* eggs (Overgaauw et al. 2009).

The prevalence of patent *Toxocara* infections in adult cats is assumed to be higher than that in adult dogs (Overgaauw 1997; Fisher 2003; Michalczyk and Sokol 2008; Gates and Nolan 2009; Overgaauw et al. 2009). Nevertheless, like in household dogs, most of the household cats are unlikely to shed *Toxocara* eggs at the moment of being dewormed blindly, i.e. without laboratory confirmation of *Toxocara* infection. *Toxocara* prevalence rates in cats vary from 2 to 79 %, depending on the country, diagnostic test, and population under study (e.g. indoor household cats, household cats with outdoor access, stray cats, sheltered cats, etc.) (Engbaek et al. 1984; Overgaauw and Boersema 1998). By burying their faeces, cats can contaminate the environment more than just superficially. Sandpits in children’s playgrounds appear to be one of the preferred spots for free-ranging cats in urban areas to defaecate, posing children at high risk of infection (Uga et al. 1996). Therefore, cats deserve more attention as a likely source of human toxocariasis (Fisher 2003).

The aim of this study was to determine the prevalence and risk factors for shedding *Toxocara* eggs in cats. Additionally, we assessed the attitudes of cat owners towards deworming.

## Material and methods

In total, 670 faecal samples from cats were coproscopically examined. These samples came from privately owned cats (*n* = 353) and from cats that were recently brought to an animal shelter (*n* = 317). Cat owners and animal handlers in the shelters participated voluntarily. Of the sheltered cats, 95 had a history of straying and 20 were recently abandoned; for 202 sheltered cats no history was provided. Parasitological examination of faecal samples was combined with epidemiological data collection using questionnaires. Faecal samples were collected during four different periods within the frameworks of four different cross-sectional studies on feline parasites: (1) from October 2010 to January 2011, (2) from June to August 2014, (3) from April to May in 2015, and (4) January to March 2016. The samples were either sent to the laboratory by the owners or by veterinarians working in a shelter or directly collected at the animal shelter by veterinary students. Every sample was processed within 4 days after defaecation.

At least 3 g of faeces per sample were examined at the parasitology laboratory of the Faculty of Veterinary Medicine of Utrecht University using the centrifugation sedimentation flotation technique. The amount of faeces was suspended in 55 ml of water and 11 ml of this suspension was used for centrifugal sedimentation followed by flotation using a sucrose solution with a specific gravity of 1.27–1.30 g/cm^3^.

Questionnaires were answered online using Surveymonkey^®^. Owners needed to complete the questionnaires to obtain the results of the coproscopical examination. Because of the different purposes of the four studies, not every question was included in the questionnaire of all studies. A copy of the questionnaire is available on request to the authors. For the sheltered cats, the animal handlers were interviewed at the animal shelter or questions were handed in paper form and returned with the samples.

### Data analysis

We assessed the association of 21 variables with positivity for *Toxocara* eggs using logistic regression models incorporating two-way cluster-robust standard errors as performed elsewhere (de Man et al. 2016) to account for clustering, i.e. non-independence, of cats at both the study (*n* = 4) and household/shelter (*n* = 395) levels. Variables showing *p* ≤ 0.10 for the association with *Toxocara* positivity in the univariable analysis were selected for inclusion in a multivariable logistic regression model built in backward stepwise fashion to retain only those variables significantly associated (*p* < 0.05) with *Toxocara* positivity. However, variables producing a change of ≥10 % in the coefficients of the other covariates when removed from the models were retained regardless of their significance. Associations were expressed as odds ratios (ORs) with corresponding 95 % confidence intervals (CIs).

Collinearities between variables were checked before multivariable analysis and choosing between collinear variables was based on the improvement in model fit (Akaike information criterion) or on biological plausibility, reliability, and number of observations when the collinear variables measured similar factors (Dohoo et al. 2009). Because of the limited number of outcome events, the final multivariable model was cross-validated by calculating bias-corrected bootstrap 95 % CIs (1000 replications) to ensure that they did not differ significantly from the standard ones, as suggested elsewhere (Vittinghoff and McCulloch 2007; Nemes et al. 2009). Statistical analysis was performed using Stata v. 13 (StataCorp., USA).

## Results

### Prevalence of gastrointestinal parasites

Of the 670 faecal samples examined for all types of helminth eggs (Table 1), 54 were found positive for at least one type of helminth egg (8.1 %, 95 % CI 6.2–10.5 %). In 49 cats, only one type of eggs was found, while 5 cats had a double infection. The most frequently found egg type was that of *Toxocara* sp. with a prevalence of 7.2 % (95 % CI: 5.4–9.4 %). As the main focus of this study was on *Toxocara*, further results were presented for this specific helminth only.

**Table 1:** Prevalence of the different helminth egg types recovered at examination of cats’ faeces

Helminths	Positive cats (*n* = 670 tested cats)	Prevalence (%)	95 % CI^a^
*Toxocara* sp.	48	7.2	5.4–9.4
*Capillaria* sp.	3	0.5	0.1–1.9
Taeniidae	7	1.1	0.5–2.2
*Toxascaris leonina*	0	0.0	0.0–0.6^b^
Hookworms	1	0.2	0.0–1.1
*Dipylidium caninum*	0	0.0	0.0–0.6^b^

*CI* confidence interval

^a^Adjusted for clustering at the levels of study cohort and household

^b^One sided, 97.5 % confidence interval

### Risk factors

The results of the univariable and multivariable analyses of the factors associated with positivity to *Toxocara* are reported in Table 2. Of the 12 factors showing a *p*  ≤ 0.10 in the univariable analysis that were selected for inclusion in the multivariable model, only 3 were significantly associated with *Toxocara* positivity in the multivariable analysis. These were cats’ age group, average daily time spent outside, and living in rural areas. Specifically, compared to cats of ≤1 year of age, those aged 2–5 years, and those aged ≥6 years had a decreased risk of being *Toxocara* positive (ORs 0.40 and 0.11, respectively). Conversely, the risk of being positive to *Toxocara* increased with the average duration of (unsupervised) outdoor time. Compared to cats that have, according to the owner, no outdoor access at all, an increased risk was found in those staying outside for an average of ≤1 h/day (OR 2.02), 2–5 h/day (OR 7.26), or ≥6 h/day (OR 8.49). Finally, cats living in rural areas were at increased risk of being *Toxocara* positive (OR 7.48).

**Table 2:** Factors associated with increased or decreased odds for positivity to *Toxocara* eggs in cats

Factor	*n*	*Toxocara* prevalence %^a^	Univariable OR^a^	Multivariable OR^a, b^
Age group				
≤1 year	36	19.4 (10–34.4)	Ref.	Ref.
2–5 years	321	7.8 (5.2–11.4)	0.35 (0.15–0.80)‡	0.40 (0.26–0.64)§
≥6 years	241	3.3 (1.7–6.4)	0.14 (0.05–0.43)§	0.11 (0.10–0.12)§
Unknown	72	11.1 (4.8–23.5)	0.52 (0.17–1.57)	0.26 (0.07–1.01)*
Gender				
Female	311	7.7 (5.3–11.2)	Ref.	
Male	336	6.8 (4.5–10.2)	0.88 (0.38–2.02)	
Unknown	23	4.3 (0.5–28.8)	0.54 (0.05–5.96)	
Time since last deworming				
≤1 month	80	8.8 (3.7–19.2)	Ref.	
2–3 months	160	5 (2.5–9.7)	0.55 (0.11–2.67)	
4–6 months	95	6.3 (2.8–13.6)	0.7 (0.15–3.32)	
≥7 months	129	2.3 (0.7–7.1)	0.25 (0.07–0.86)†	
Unknown	206	11.7 (7.7–17.3)	1.38 (0.62–3.05)	
Applied deworming regimen				
None	91	1.1 (0.2–7.6)	Ref.	
1 times/year	42	0.0 (0.0–8.4)^d^	Not estimable	
2–3 times/year	120	2.5 (0.8–7.6)	2.31 (0.22–23.7)	
≥4 times/year	82	1.2 (0.2–7.8)	1.11 (0.07–17.68)	
Unknown	335	12.8 (9.4–17.3)	13.25 (1.75–100.12)†	
Sterilization				
No	283	12.7 (9–17.6)	Ref.	
Yes	364	3 (1.7–5.3)	0.21 (0.14–0.33)§	
Unknown	23	4.3 (0.5–28.8)	0.31 (0.05–2.06)	
Outdoor access				
No	254	2.4 (1.1–5.1)	Ref.	
Yes	297	8.1 (5.4–12)	3.63 (2.15–6.15)§	
Unknown	119	15.1 (9.3–23.6)	7.37 (1.58–34.41)‡	
Average daily time spent outdoor				
None (no outdoor access)	254	2.4 (1.1–5.1)	Ref.	Ref.
≤1 h	28	7.1 (1–37.4)	3.18 (0.87–11.66)*	2.02 (1.08–3.75)†
2–5 h	35	22.9 (12.4–38.2)	12.25 (3.34–44.89)§	7.26 (3.82–13.79)§
≥6 h	18	27.8 (11.7–52.7)	15.90 (4.34–58.28)§	8.49 (4.89–14.74)§
Unknown outdoor hours	216	4.2 (2.2–7.8)	1.80 (0.54–5.96)	1.09 (0.4–2.92)
Unknown outdoor access	119	15.1 (9.3–23.6)	7.37 (1.58–34.41)‡	1.70 (0.56–5.19)
Urban area^c^				
No	381	3.9 (2.4–6.5)	Ref.	
Yes	100	9 (4.5–17.3)	2.41 (1.46–3.99)§	
Unknown	189	12.7 (8.3–19)	3.55 (0.58–21.73)	
Woody area^c^				
No	468	4.7 (3.1–7.1)	Ref.	
Yes	13	15.4 (3.6–46.9)	3.69 (0.41–33)	
Unknown	189	12.7 (8.3–19)	2.95 (0.47–18.56)	
Rural areas^c^				
No	448	2.9 (1.7–5)	Ref.	Ref.
Yes	33	33.3 (19.2–51.2)	16.73 (4.77–58.71)§	7.48 (2.4–23.35)§
Unknown	189	12.7 (8.3–19)	4.87 (0.94–25.12)*	5.39 (2.47–11.8)§
Feeding raw meat				
No	314	6.7 (4.3–10.2)	Ref.	
Yes	170	2.4 (0.9–6.1)	0.34 (0.13–0.88)†	
Unknown	186	12.4 (8–18.7)	1.97 (0.37–10.53)	
Feeding raw fish				
No	400	5.5 (3.6–8.3)	Ref.	
Yes	81	2.5 (0.6–9.5)	0.43 (0.06–3.24)	
Unknown	189	12.7 (8.3–19)	2.50 (0.39–15.85)	
Predation				
No	452	4.4 (2.8–6.9)	Ref.	
Yes	87	11.5 (6.2–20.3)	2.81 (0.81–9.66)*	
Unknown	131	13.7 (8.3–21.9)	3.44 (0.44–27.03)	
Sheltered in the last 6 months				
No	441	4.1 (2.5–6.6)	Ref.	
Yes	40	15 (7.5–27.6)	4.15 (1.16–14.85)†	
Unknown	189	12.7 (8.3–19)	3.42 (0.58–20.09)	
Preferential defaecation				
Indoor (litterbox)	370	2.4 (1.2–4.9)	Ref.	
Outdoor	29	27.6 (15.8–43.6)	15.28 (9.61–24.3)§	
Both indoor and outdoor	75	8 (3.7–16.6)	3.49 (1.41–8.62)‡	
Unknown	196	12.8 (8.4–18.9)	5.86 (1.21–28.35)†	
Frequency of litterbox cleaning				
No litterbox	39	23.1 (13.5–36.5)	Ref.	
≤1 times/week	32	9.4 (3–25.8)	0.34 (0.12–1.03)*	
2 times/week	69	2.9 (0.7–10.8)	0.1 (0.06–0.17)§	
≥3 times/week	341	2.9 (1.5–5.6)	0.1 (0.08–0.12)§	
Unknown	189	12.7 (8.3–19)	0.48 (0.11–2.16)	
Diarrhoea				
No	431	4.9 (3.2–7.4)	Ref.	
Yes	50	6 (1.4–21.9)	1.25 (0.32–4.91)	
Unknown	189	12.7 (8.3–19)	2.84 (0.58–13.9)	
Discoloration in the stool				
No	470	4.9 (3.2–7.4)	Ref.	
Yes	11	9.1 (1.2–44.8)	1.94 (0.39–9.64)	
Unknown	189	12.7 (8.3–19)	2.83 (0.52–15.34)	
Gastrointestinal conditions				
No	226	9.3 (5.9–14.4)	Ref.	
Yes	53	5.7 (1.8–16.2)	0.59 (0.06–5.41)	
Unknown	391	6.1 (4.2–8.9)	0.64 (0.13–3.12)	
Cardiological and/or respiratory conditions				
No	246	8.9 (5.7–13.7)	Ref.	
Yes	33	6.1 (1.8–18.7)	0.66 (0.05–9.19)	
Unknown	391	6.1 (4.2–8.9)	0.67 (0.13–3.37)	
Nephrological and/or metabolic conditions				
No	251	9.2 (6–13.8)	Ref.	
Yes	28	3.6 (0.5–21.7)	0.37 (0.02–6.99)	
Unknown	391	6.1 (4.2–8.9)	0.65 (0.12–3.53)	

*p ≤ 0.10; †*p* < 0.05; ‡*p* ≤ 0.01; §*p* ≤ 0.001

*OR* odds ratio

^a^Adjusted for clustering at the levels of study cohort and household

^b^Adjusted for all variables whose ORs appear in this column

^c^The living environment was reported by the owners based on the prevalent characteristics of their neighbourhood as suggested by the questionnaire; an urban (residential) area was defined as the one containing mainly paved roads, sidewalks, and houses with small or no green areas; a rural area contained few trees but mainly pastures and meadows; and a woody areas consisted mainly of forests and shrubs

^d^One sided, 97.5 % confidence interval

### Owner’s attitude towards deworming

Of the 335 cats tested for *Toxocara* and for which the deworming regimen was reported, 91 (27.2 %) had never received an anthelmintic treatment according to the owner, 42 (12.5 %) were treated at least once a year, 120 (35.8 %) 2–3 times a year, and 82 (24.5 %) ≥4 times a year. The frequency of treatment was not significantly associated with *Toxocara* positivity (Table 2). Of the 464 cats tested for *Toxocara* and for which the time since last deworming was known, 80 (17.2 %) had received an anthelminthic treatment within 1 month before sampling, 160 (34.5 %) between 1 and 3 months, 95 (20.5 %) between 4 and 6 months, and 129 (27.8 %) more than 6 months before. The time of last deworming did not have a significant effect on the risk of being *Toxocara* positive (Table 2). There was no significant relation between the time the cat spends outdoors and the frequency of deworming.

Information on the main reasons for anthelmintic treatment was provided for 199 cats, corresponding to 81.6 % of the cats for which a deworming regimen was implemented. The “cat’s health” was the main reason to deworm 160 cats (80.4 %), followed by “public health” (21 cats, 10.6 %), “because we must” (9 cats, 4.5 %), and a combination of these (9 cats, 4.5 %). There was no significant association between the main reason for deworming and the applied deworming frequency.

## Discussion

Although infections with endoparasites are generally less studied in cats than in dogs, there are several reports on the prevalence of patent infections with *T. cati* in cats that indicate that cats are responsible for a considerable part of the environmental contamination with this zoonotic roundworm (Fisher 2003). In the Netherlands, the number of household cats exceeds the number of household dogs (HAS den Bosch and Utrecht University 2015) and, while there are no stray dogs, there is a large stray cat population (Neijenhuis and van Niekerk 2015). This, combined with the typical feline defaecation behaviour, leads to cats being responsible for a substantial contribution to the environmental contamination with *Toxocara* eggs and possibly the occurrence of toxocariasis in humans (Nijsse et al. 2015). Therefore, the public health relevance of *T. cati* should not be underestimated (Fisher 2003).

With an overall prevalence of 7.2 %, cats in the Netherlands appear to be moderately infected with *T. cati* as compared to the mean European prevalence of 19.7 % reported in 2014 (Beugnet et al. 2014). Our prevalence is lower than the one of 28.2 % reported in 2004 among sheltered cats in the Netherlands (Robben et al. 2004), but it is comparable with prevalence rates in Germany (4.7–6.4 %) (Barutzki and Schaper 2003; Barutzki and Schaper 2011) and the USA (7.5 %) (Gates and Nolan 2009). However, it is much lower than the prevalence rates in areas that have comparable settings to the Netherlands, like Belgium, the northern part of Germany, and Denmark, with reported prevalences of 60 % (Vanparijs et al. 1991), 27.1 % (Becker et al. 2012) and 79 % (Engbaek et al. 1984), respectively. The difficulty in comparing these prevalence rates derives from the different lifestyles within household cat populations and the concomitant differences in exposure to common risk factors. In Mexico City, the prevalence in apartment cats was only half of that found in other household cats; however, both these prevalences (20.7 and 42.5 %, respectively) (Martinez-Barbabosa et al. 2003) were higher than that found in this study. Extrapolating our results to the overall cat population, which is generally more heterogeneous than the one under study here, is challenging. For instance, in our study cats with restricted outdoor access and cats under proper veterinary care were probably overrepresented, which possibly led to an underestimation of the real *Toxocara* prevalence in the overall population.

Studies focussing on risk factors for helminth infections in cats are scarce (Mircean et al. 2010; Beugnet et al. 2014). In our study, significant risk factors in the multivariable analysis were young age, living in rural areas, and roaming freely outdoors. Age is a known risk factor for ascarid infections of dogs and cats, which can be partially explained by the development of a functional immunological response. However, age resistance in household cats is probably less effective in preventing patent infections than in household dogs due to the predatory behaviour of cats (Overgaauw and van Knapen 2013). Age as a risk factor for cats was also described for cats in other studies (Mircean et al. 2010; Barutzki and Schaper 2011; Beugnet et al. 2014). The standard deworming advice for kittens states that they should be dewormed every 2 weeks from the age of 3 weeks until they are 8 weeks of age, followed by monthly deworming up to 6 months of age (ESCCAP 2010). From that age on, age resistance is expected to prevent the development of patent infections. However, our data and those of other studies conclude that cats are at higher risk of developing patent infections up to 1 year of age.

Increasing time spent outdoors is a known risk factor for *Toxocara* infection in cats (Beugnet et al. 2014) and we observed an outdoor time-dependent relation with the risk of *Toxocara* infections, meaning that the more time a cat spends outside (unsupervised) the greater the risk of developing a patent infection. This may be related to the chance of ingesting infective eggs from the environment, but likely also to more time spent predating. However, predation itself did not prove to be a significant risk factor in the multivariable analysis. The reported predatory behaviour, however, is a reflection of what was observed by the owner/caregiver. When a cat is outside without supervision, the predatory behaviour cannot always be witnessed with certainty, and unnoticed consumption of paratenic hosts might lead to patent infections. Living in a rural area is probably mirroring a higher chance for cats to encounter infective stages of *Toxocara*, either in the environment or in prey. Farm cats are usually free to roam in the surroundings and they are commonly a part of a farm’s pest control plan by catching small rodents. The relation between living in rural areas and being at risk of developing patent *Toxocara* infections was also described by Mircean et al. (Mircean et al. 2010). Stray cats probably spend even more time unattended outside, exposed to the same factors, but are likely lacking any preventative veterinary care. Therefore, their contribution to environmental contamination with *Toxocara* eggs is assumed to be considerable (Fisher 2003; Morgan et al. 2013; Nijsse et al. 2015).

The lack of a significant association between the time since last deworming and patent *Toxocara* infection is surprising and needs to be further investigated. We also found that the advised deworming frequency of cats of at least four times a year was applied by 24.5 % of the cat owners who reported their treatment regimen, meaning that 75.5 % of those cat owners dewormed their animal less frequently. Most cat owners (80.4 %) answering the question about the main reason for deworming their cats answered to do this because of their cats’ health and only 10.6 % answered that the primary reason was “public health”. Both deworming frequency and incentive for deworming show that owners are not aware, and possibly misinformed, about why deworming is necessary. This remains a point of attention as reported before (Overgaauw and Boersema 1996; Overgaauw et al. 2009; Nijsse et al. 2014). A more custom-made deworming advice with attention for the risk factors of an individual cat could convince an owner to pay more attention to the deworming strategy of their cats. This is already propagated by ESCCAP as an alternative for blind deworming. A risk-based decision tree has been made available (ESCCAP 2014). This is still a very coarse tool, but it can help owners and veterinarians in assessing the risk group their cat fits into. Studies like ours can help to refine such tools for veterinarians and cat owners.

In conclusion, our results show that about 7 % of cats in the Netherlands shed *Toxocara* eggs. Besides young age and living in rural areas, we found that the more time a cat spends outdoors, the higher the risk for this cat to shed *Toxocara* eggs, indicating that stray and free-roaming cats are more likely to contaminate their living environment with *Toxocara* eggs. The overall 7.2 % prevalence in cats is higher than that observed in household dogs in the Netherlands (Nijsse et al. 2014). In conjunction with the fact that there are more cats than dogs, this implies that cats should receive more attention as a source of *Toxocara* eggs in the environment. Moreover, insufficient knowledge on the zoonotic aspects of *Toxocara* in combination with the low compliance to the advice of routinely deworming cats stresses the importance of educating cat owners about this parasitic infection of cats, the zoonotic risk, and the rationale of following a (preferably risk based) deworming regimen.
